# An Atypical Case of Acrokeratosis Verruciformis of Hopf Responding to Combined Acitretin and Cryotherapy

**DOI:** 10.7759/cureus.60000

**Published:** 2024-05-09

**Authors:** Aniket Goswami, Anita Marak, Shikha Verma, Biswajit Dey

**Affiliations:** 1 Dermatology, North Eastern Indira Gandhi Regional Institute of Health and Medical Sciences, Shillong, IND; 2 Pathology, North Eastern Indira Gandhi Regional Institute of Health and Medical Sciences, Shillong, IND

**Keywords:** acitretin, cryotherapy, atp2a2, church spires, acrokeratosis verruciformis of hopf

## Abstract

Acrokeratosis verruciformis of Hopf (AKVH) is a rare genetic skin condition associated with an *ATP2A2* gene mutation, thus affecting keratinization. Classically, AKVH appears in childhood over acral sites as symmetrical, flat, verruca plana-like lesions with an autosomal dominant inheritance, while sporadic cases affect atypical sites in adulthood. As this entity can closely mimic other verrucous skin conditions, identifying characteristic histopathological changes is essential to make a diagnosis in the absence of genetic studies, especially in resource-poor countries. This is the first reported case of AKVH from North-East India clinically mimicking extensive verruca vulgaris in an adult with a possible sporadic occurrence. AKVH is usually difficult to treat and superficial ablation is the treatment of choice. However, this case highlights the role of cryotherapy with acitretin in the management of AKVH with a rapid response.

## Introduction

In 1931, Dr. Gustav Hopf described acrokeratosis verruciformis of Hopf (AKVH), which poses a unique dermatological challenge due to its clinical similarities with conditions like epidermodysplasia verruciformis, verruca plana, and Darier’s disease. AKVH, characterized by keratotic lesions on the distal part of the extremities, stems from an autosomal dominant inheritance pattern associated with an *ATP2A2* gene mutation. Typically manifesting from birth to early childhood with familial links, sporadic cases may emerge in the fifth decade without inclination towards any particular sex [1].

Clinically, AKVH displays flat-topped keratotic papules and plaques on hands and feet, often with nail changes. Driven by the P602L mutation in *ATP2A2*, which results in disordered keratinization, it thus leads to a chronic, recurrent course and the potential risk of malignant transformation [1].

Superficial ablation, notably effective, offers a viable therapeutic approach, although recurrence remains a consideration with alternative methods like cryotherapy and surgical excision with less than satisfactory end results [2].

We report a sporadic case of AKVH with extensive giant verrucous cauliflower-like lesions where improvement was noted with concurrent use of acitretin and cryotherapy, as superficial ablation was not feasible due to extensive involvement of the affected sites.

## Case presentation

**Figure 1:**
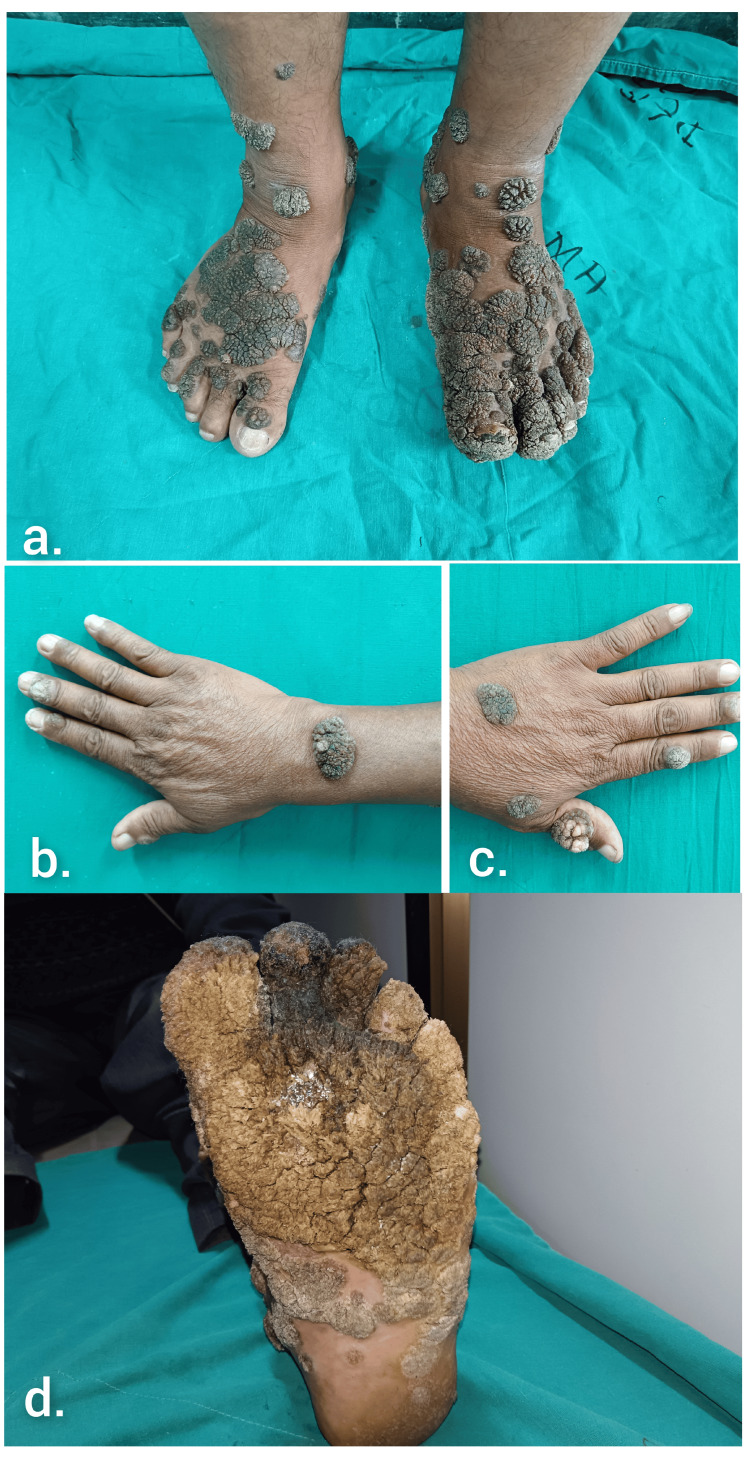
Hyperkeratotic, cauliflower-like giant verrucous plaques over (a) dorsa of both feet, (b) right hand, (c) left hand, and (d) plantar aspect of left foot

A 50-year-old male driver, with type 2 diabetes mellitus on treatment, presented with extensive verrucous proliferative growth of insidious onset over his bilateral hands and feet for 20 years associated with pain and difficulty in walking for the past two months. The patient denied history of any trauma or exposure to any environmental pollutants. Also, there was no history of similar illness in the family. Cutaneous examination revealed multiple discrete to coalescent hyperpigmented, hyperkeratotic, cauliflower-like giant verrucous plaques on the dorsal aspect of bilateral hands, feet, and plantar aspect of the left foot (Figure 1). Dystrophic nail changes in the form of nail plate thickening and discoloration were noted over the left toenails. Other body sites were not affected. Excisional biopsy was done from the right-hand lesion with the differentials of verruca vulgaris, epidermodysplasia verruciformis, and to rule out underlying squamous cell carcinoma. 

**Figure 2:**
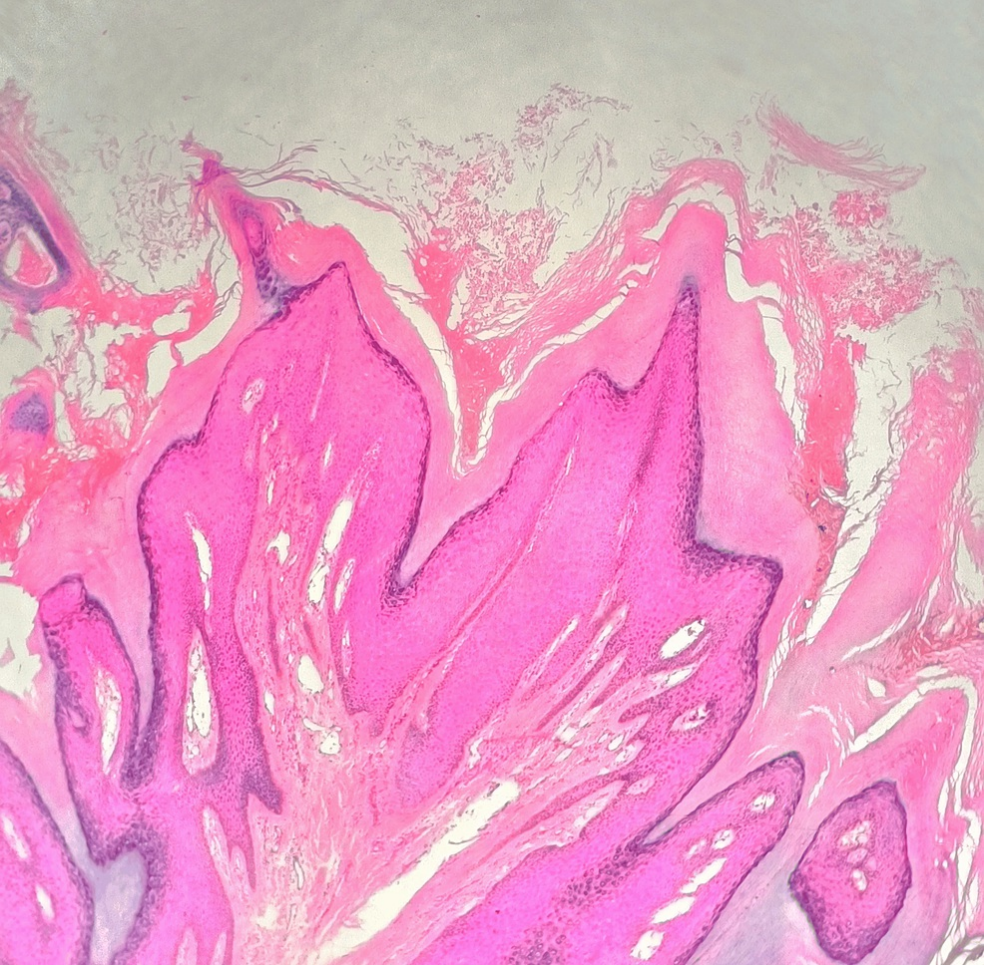
Histopathology showing hyperkeratosis, hypergranulosis, and acanthosis with marked papillomatosis without vacuolization resembling "church spires" (H & E, 200x)

Histopathological examination revealed hyperkeratosis, hypergranulosis, acanthosis, marked papillomatosis resembling "church spires" (Figure 2), and peri-vascular with peri-adnexal chronic inflammatory cell infiltrates. There was neither any evidence of large cells with blue-grey cytoplasm nor any koilocytes. Malignancy was ruled out due to the absence of any dysplastic or atypical malignant changes. Thus histopathologically, features were in favour of AKVH

**Figure 3:**
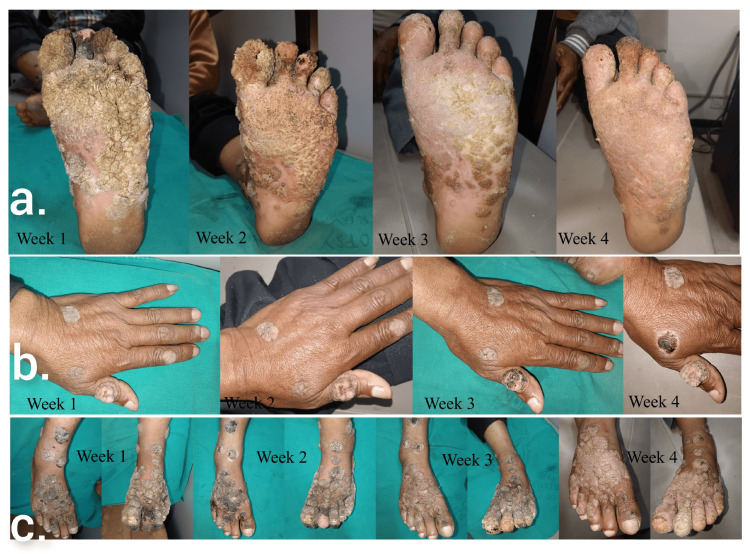
Treatment response to combined oral acitretin and cryotherapy over four weeks in (a) plantar aspect of left foot, (b) left hand, and (c) dorsal aspect of both feet

As part of the management plan, baseline investigations were done including complete blood counts, liver function test, renal function test, fasting blood sugar, glycated hemoglobin (HbA1c), and fasting lipid profile which were found to be within normal limits. The patient was started on a once-daily dose of 25 mg acitretin orally along with a once-weekly course of cryotherapy which was administered as two freeze-thaw cycles of 5-10 seconds each over individual lesions. Marked improvement was noted over a period of four weeks with flattening out of lesions (Figure 3). Response to treatment was clinically more evident over the feet lesions as compared to the hand lesions. Post-cryotherapy pain was managed with oral acetaminophen on an SOS (si opus sit; if needed) basis. The patient was in remission and he was advised for monthly follow-up to look for any evidence of recurrence.

## Discussion

AKVH is a rare hyperkeratotic genodermatosis, which was first identified by a German dermatologist, Hopf, in 1931 and exhibits a chronic course with an autosomal dominant inheritance pattern. It often presents as multiple, symmetrical, flat, skin-colored plane wart-like lesions, primarily on the hands and feet [1]. Although our case only had acral involvement, the extensive giant verrucous features were against the clinical possibility of AKVH but closely mimicked verruca vulgaris.

Classical AKVH typically manifests during childhood, affecting the hands and feet. In contrast, sporadic AKVH, occurring towards the fifth decade of life, may involve atypical sites like the face, scalp, and trunk [3]. However, unlike a case reported by Diwan et al. [4], our case of sporadic AKVH did not present with any lesions over atypical sites. Family history, palmar pits, and nail changes are characteristic of classical AKVH, nonetheless, nail changes were also noted in our case.

Classic histopathological features of AKVH include hyperkeratosis without parakeratosis, acanthosis, and circumscribed epidermal elevations or papillomatosis without vacuolization, resembling a church spire within wavy stratum corneum concavities [5,6]. These features were also evident in our case. Corps ronds, which is a feature of Darier's disease was not found in our histopathological study. The absence of koilocytes and distinctive "blue cells" with pale cytoplasm and basophilic keratohyalin granules indicative of human papillomavirus infection ruled out the other possibilities like verruca vulgaris and epidermodysplasia verruciformis [7]. Thus, histopathological evaluation is of paramount importance in diagnosing this condition, especially if genetic evaluation is not possible.

Superficial ablation is a recognized and effective treatment for AKVH while other methods include cryotherapy, laser therapy, and surgical excision. Despite attempts with retinoids, cryotherapy, or lasers, only superficial ablation is deemed effective for AKVH. Even so, lesions persist and recur, becoming more pronounced with prolonged sun exposure [1-3]. Hence, patients are required to be closely followed up to look for recurrence as was advised in our case but this is limited by various patient-related factors. Notwithstanding, in our case, superficial ablation was not favored due to extensive lesions, and cryotherapy along with oral acitretin was preferred. Andrade et al. reported improvement of lesions with the use of oral acitretin [8], as was also noted in our case. The role of combined acitretin and cryotherapy in the management of AKVH, however, has rarely been reported in the literature.

## Conclusions

To the best of our knowledge, this is the first reported case of a sporadic variant of AKVH from North-East India. This case reiterates the fact that clinicians should consider the possibility of AKVH in cases of extensive verruca vulgaris, especially over acral sites, and carry out a histopathological evaluation in all doubtful cases. Also, alternative management plans like a combined use of cryotherapy and oral acitretin should be taken into consideration, especially if superficial ablation is not feasible.
